# Comparison of hormonal receptor expression and HER2 status between circulating tumor cells and breast cancer metastases

**DOI:** 10.6061/clinics/2021/e2971

**Published:** 2021-09-28

**Authors:** Solange Moraes Sanches, Alexcia Camila Braun, Vinicius Fernando Calsavara, Paula Nicole Vieira Pinto Barbosa, Ludmilla Thome Domingos Chinen

**Affiliations:** I Departamento de Oncologia Clinica, A.C. Camargo Cancer Center, Sao Paulo, SP, BR.; II Centro Internacional de Pesquisa, A.C. Camargo Cancer Center, Sao Paulo, SP, BR.; III Departamento de Imagem, A.C. Camargo Cancer Center, Sao Paulo, SP, BR.

**Keywords:** Circulating Tumor Cells, Breast Cancer, Hormone Receptor

## Abstract

**OBJECTIVES:**

Breast cancer (BC) is the most common neoplasm in women. Biopsy of metastatic lesions is recommended to confirm estrogen receptor (ER), progesterone receptor (PR), and human epidermal growth factor receptor 2 (HER2) status as there are discrepancies in these patterns between primary tumors and metastases in up to 40% of the cases. Circulating tumor cells (CTCs) are related to BC outcomes and could potentially be an alternative to the invasive procedures of metastasis rebiopsy. ISET^®^ technology is not currently employed to detect CTCs in patients with BC. Emerging data support that the characterization of CTC protein expression can refine its prognostic value. Transforming growth factor (TGF)-β plays a role in BC progression and invasiveness. Thus, in this study, we aimed to compare ER, PR, and HER2 expression in primary tumors, CTCs, and metastases and evaluate TGF-β type 1 receptor (TGF-β RI) expression in CTCs as prognostic factor for progression free survival (PFS) and overall survival (OS).

**METHODS:**

This prospective study was conducted at the A.C. Camargo Cancer Center, Brazil. Blood samples were processed in ISET^®^ (Isolation by SizE of Tumors, Rarecells, France) before computed tomography-guided biopsy of suspected metastatic lesions. Protein expression levels in CTCs were compared to those in primary tumors/metastases (medical records).

**RESULTS:**

Of the 39 patients initially included, 27 underwent both biopsies of metastases and blood collection and were considered for analysis. The concordance rates for ER, PR, and HER2 expression between primary tumors and metastases were high. No loss of HER2 expression at any metastasis site and retention of the same pattern of protein expression in all triple-negative (TN) tumors (92.5%, 81.5% and 96.2% respectively) (*p*<0.0001) was observed. When metastases/CTCs were classified as TN/non-TN, CTCs showed high specificity (93%), accuracy (84.2%), and negative predictive value (88%). The median OS of patients without TGF-β RI expression in CTCs was 42.6 *versus* 20.8 months for TGF-β RI expression-positive ones (*p*>0.05).

**CONCLUSION:**

The role of CTCs detected by ISET has not yet been established in BC. Here, we suggest that this methodology may be useful to evaluate metastasis in non-TN cases as well as TGF-β RI expression in CTCs, which may impact patient survival. Due to sample limitations, future studies must focus on specific BC subtypes and an expansion of the cohort.

## INTRODUCTION

Breast cancer is the most common neoplasm in women, and it continues to be associated with a high mortality rate despite the increasing number of early diagnoses and improvement of the initial tumor cure rate (1). Breast cancer is metastatic at the time of diagnosis in approximately 6-10% of the patients, and 20-30% of the patients develop metastasis throughout their disease course (2). The treatment of metastatic breast cancer is palliative and must be efficient and preserve the patients’ quality of life.

When safe and accessible, a biopsy of metastatic breast cancer lesions is recommended to confirm the diagnosis, determine the hormone receptor (HR) and human epidermal growth factor receptor 2 (HER2) status, and potentially guide treatment. Discrepancies in the patterns of breast cancer markers between primary tumors and metastatic lesions, reflecting changes in tumor biology, occur in up to 40% of the cases (3-5) and result in changes in therapy in approximately 14% of the cases (6).

As tumor evolution is dynamic, determination of the metastatic phenotype via repeated sampling provides real-time information about molecular changes, enabling further treatment customization. However, the need for repeated invasive procedures limits this approach. Thus, identifying agile and less invasive tools that could provide useful information for therapeutic decision making is needed.

Circulating tumor cells (CTCs) can be isolated from the blood of patients with breast cancer using several different methods (7,8). The prognostic impact of CTC assessment has been demonstrated in several studies. The presence of CTCs in the blood of patients with metastatic breast cancer and the variability in the number of these cells over the treatment course correlates with tumor progression and overall survival (9,10). Changes in treatment directed by the number of CTCs have not been found to change the clinical evolution of the disease (11), but a recent trial suggested CTCs as a biomarker to guide first-line therapy in ER^+^ BC (12). Distinct aggressive behaviors have also been observed in patients with HR-positive metastatic breast cancer, depending on the number of CTCs present (13). In patients with non-metastatic disease, the presence of CTCs at the time of surgery (14,15) or shortly after the end of adjuvant chemotherapy (16) is predictive of early recurrence and reduced overall survival. Furthermore, the presence of CTCs in patients without evidence of active disease, even at five years after breast surgery, was correlated with higher recurrence rates over a median period of 2.8 years (17). A recent study demonstrated the utility of CTC data for clinical decision making (12), and ongoing studies have considered not only the number but also the phenotype of CTCs to guide treatment (18).

The guidelines of the National Comprehensive Cancer Network recommend the consideration of CTCs in breast cancer staging, defining cM0 (i+) as “no clinical or radiographic evidence of distant metastasis, but cells or cell deposits smaller than 0.2 mm detected microscopically or via molecular techniques in circulating blood, bone marrow, or other non-regional lymph node tissue in a patient without symptoms or signs of metastasis.” These guidelines do not include CTC assessment in the evaluation or monitoring of metastatic disease, although the prognostic capacity of such assessments is recognized (19).

Several techniques have been used to isolate and characterize CTCs. The most frequently used system is CellSearch^®^ (Menarini Silicon Biosystems), which is based on immunomagnetic separation and uses epithelial cell adhesion molecule (EpCAM) as an indicative of epithelial cells. The ISET^®^ technology (Rarecells Diagnostics, Paris, France) isolates cells by size through membranes with 8.0 μm pores, independent of tumor markers, which enables the immunocytochemical characterization of cells (20). A study that compared CTC detection between the CellSearch^®^ and ISET^®^ systems showed less detection of cell lines with lower EpCAM expression by the former, suggesting that CellSearch^®^ does not adequately identify cells in the epithelial-mesenchymal (EMT) transition phase (8). In another study, the findings of CellSearch^®^ and ISET^®^ were concordant in 55% of cases of metastatic breast cancer, 60% of cases of prostate cancer, and 20% of lung cancer cases (21), confirming the limitation of the EpCAM-dependent method.

During EMT, molecules such as the dual-functioning transforming growth factor (TGF)-β act as growth-inhibiting cytokines in normal epithelial cells and primary breast tumors. They also play a role in cancer progression, tumor cell invasiveness and metastases, modifying the tumor microenvironment, and promoting EMT (22,23).

Considering CTC detection as a “liquid biopsy,” comparison of the protein expression profiles of CTCs and metastases would be of interest to determine whether CTC data can be used as a surrogate for metastatic site biopsy findings. Hence, the objectives of this study were to compare estrogen receptor (ER), progesterone receptor (PR), and HER2 expression in three tumor compartments (primary tumors, CTCs, and metastases) in patients with breast cancer and to evaluate TGF-β type 1 receptor (TGF-β RI) expression in CTCs as a prognostic factor correlated with the progression-free and overall survival of these patients.

## MATERIALS AND METHODS

### Patients and study design

This study was approved by the ethics committee (CEP 2345/17) of our institute (A.C. Camargo Cancer Center) and was included two cohorts of breast cancer patients treated at the A.C. Camargo Cancer Center, São Paulo, Brazil. Patients treated between September 2017 and July 2019 and a retrospective cohort treated between October 2013 and January 2015 were included (samples from the patients of an unpublished study). Both cohorts had clinical indications for biopsy because of suspicious lesion imaging findings. The included patients were aged >18 years, and the extent of their disease was measured using the Response Evaluation Criteria in Solid Tumors (version 1.1.34). Patients who had undergone bone biopsy or had a history of other neoplasms were excluded.

Blood samples were collected immediately before the computed tomography-guided biopsy of the suspected metastatic lesions. ER, PR, and HER2 protein expression levels in CTCs were compared to those in primary tumors and metastases using data obtained from medical records. Biopsy samples were considered ER- and PR-positive if 1% to 100% of the tumor nuclei were stained via immunohistochemistry, and HER2-positive if complete membrane staining in more than 10% of cells was strong (3+) or amplified by *in situ* hybridization in weak to moderate staining (2+), according to the updated American Society of Clinical Oncology/College of American Pathologists (ASCO/CAP) guidelines (24,25). The clinical and pathological characteristics of the patients were also obtained from medical records.

### Isolation of CTCs

Just before the scheduled biopsy of a suspected metastasis, 8 mL of blood was collected from each patient via peripheral venipuncture, placed in EDTA tubes (BD Vacutainer^®^), and homogenized for up to 4h at room temperature (18-23°C) until processing.

CTCs were isolated via filtration, through a policarbonate membrane, that is a component of ISET system, using differences in the size of epithelial tumor cells, according to the manufacturer’s instructions (ISET^®^; Rarecells Diagnostics). After filtering, the membranes were washed with phosphate-buffered saline (PBS), dried in open air overnight, and stored at -20°C until analysis.

### Immunocytochemistry

Immunocytochemical assays for ER, PR, HER2, and TGF-^®^ RI were performed using ISET^®^ membranes. Briefly, the membranes containing the captured CTCs were cut and placed in 24-well plates for antigenic recovery, followed by hydration.

The cells were permeabilized with Triton 0.2% in phosphate-buffered saline (PBS), and endogenous peroxidase was blocked with 3% hydrogen peroxide. The membrane spots were then subjected to double-labeled immunocytochemistry. The following antibodies were used: anti-epidermal growth factor receptor (1:500 dilution, batch GR3215639-3; Abcam, Cambridge, United Kingdom), anti-HER2 (1:400 dilution, batch 4290S; Cell Signaling Technologies, Danvers, MA, USA), anti-estrogen receptor (1:300 dilution, batch SL2494175A; Invitrogen, Carlsbad, California, USA), anti-TGF-β RI (1:500 dilution, lot 3066103; Merck, Darmstadt, Germany), and anti-progesterone receptor (1:100 dilution, lot G02275; CusAb, Houston, Texas, USA). To confirm that the analyzed circulating cells were not leukocytes, we used an anti-CD45 antibody (1:100 dilution, lot F1222Y; CusAb). All antibodies were separately diluted in PBS and 10% fetal bovine serum. To amplify the antibody signals, the spots were incubated with the Envision G/2 Doublestain rabbit/mouse system (Agilent Technologies), followed by incubation with DAB and permanent red (Agilent Technologies). The cells were then stained with hematoxylin and analyzed under an optical microscope (BX61; Olympus, Tokyo, Japan).

CTCs were identified using the following criteria: negative CD45 staining, hyperchromatic and irregular nuclei ≥12 μm in diameter, visible cytoplasm, and high nucleus/cytoplasm ratio (0.8) (26).

CTC counts were determined as the number of CTCs per milliliter of blood, defined as one spot on the ISET^®^ membrane. CTC results were considered positive when at least one of the four ISET^®^ spots analyzed per patient contained a CTC (at least one CTC in 4 mL blood or 0.25 CTC in 1 mL blood) (26). We recorded positive ER, PR, HER2, and TGF-β RI expression in CTCs, regardless of the intensity. It is important to emphasize that sometimes, even when the patient has many CTCs, some ISET spots show no CTCs. Therefore, when the spot had no CTCs, it was not possible to evaluate protein expression.

### Statistical analysis

Baseline patient characteristics were expressed as absolute and relative frequencies for qualitative variables and as medians and ranges for quantitative variables. Associations between qualitative variables were evaluated using the chi-square test or Fisher’s exact test, as appropriate. Associations between continuous and qualitative variables were evaluated using the Mann-Whitney U test. Sensitivity, specificity, negative and positive predictive values, and accuracy were also calculated. The kappa statistic was used to characterize the degree of concordance of protein expression among primary tumors (including subtypes), metastases, and CTCs. Survival curves were estimated using the Kaplan-Meier estimator, and differences between the curves were assessed using the log-rank test. Regarding the age at diagnosis, the determination of two groups of observations with respect to a simple cut-off point was estimated using the maximum of the standardized log-rank statistic proposed by Lausen and Schumacher (27). Progression-free survival and overall survival were measured from the time of CTC collection until disease progression, as determined via imaging studies. Patients who experienced no events were censored at their last visit to the hospital or death. The significance level was fixed at 5% for all tests. Statistical analyses were performed using IBM SPSS Statistics (version 24.0; IBM Corporation, Armonk, NY, USA) and R software (version 3.4; R Foundation for Statistical Computing, Vienna, Austria).

## RESULTS

**Figure 1:**
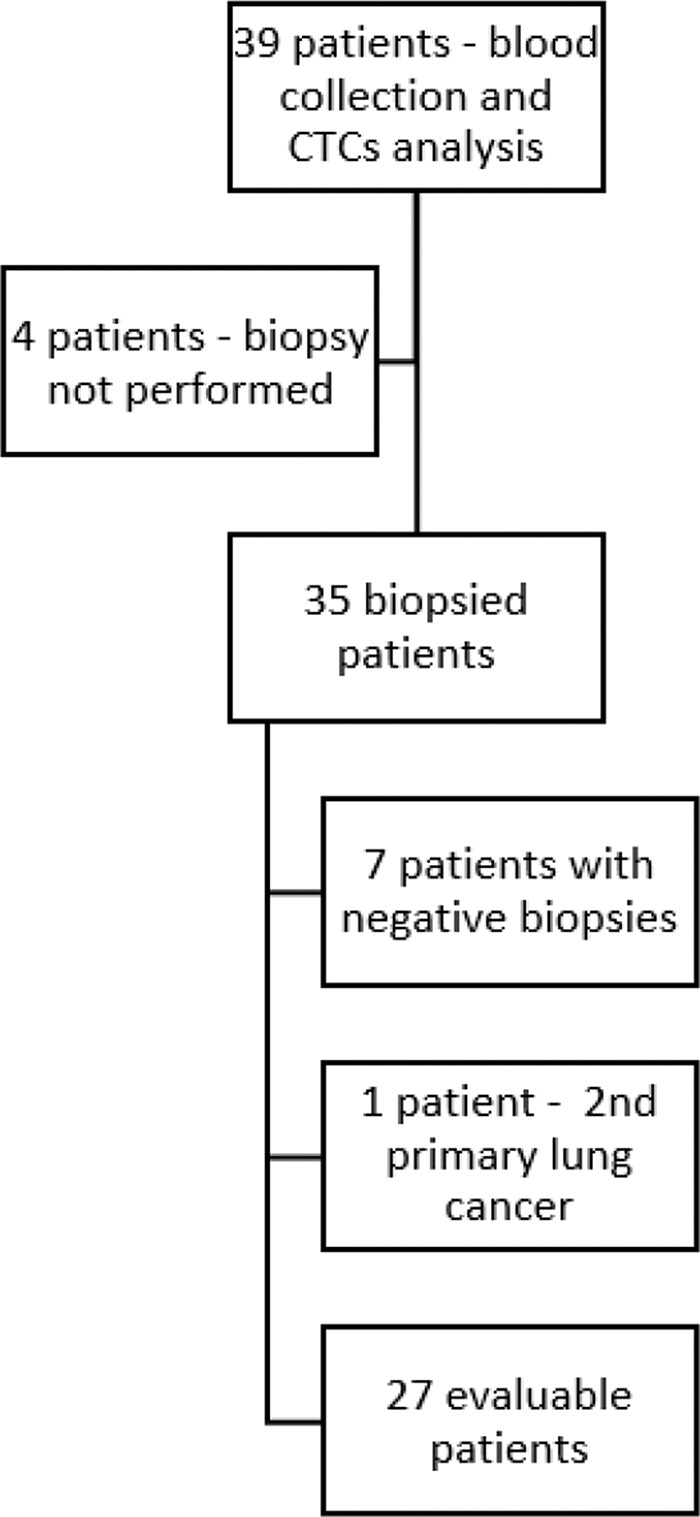
Flowchart showing patient inclusion, blood collection, biopsies, and reasons for excluding patients. **Abbreviations:** CTCs (circulating tumor cells).

In total, 39 patients with breast cancer were included in the study (30 patients from the prospective cohort and 9 patients from the retrospective cohort). Of these, four patients did not undergo a biopsy and were excluded. The remaining 35 patients underwent a biopsy of lesions suspected to be metastases, with blood collection for CTC assessment performed immediately before the procedures. Seven biopsies revealed no evidence of neoplastic cells. New primary lung cancer was diagnosed in one patient, resulting in 27 patients with both biopsies of metastases and blood collection considered for analysis (Figure 1). The median age of the patients was 39 years (63% had cancers of the luminal type). The median follow-up duration was 20.8 months. The clinical and pathological characteristics of the patients are summarized in Table 1.

**Figure 2:**
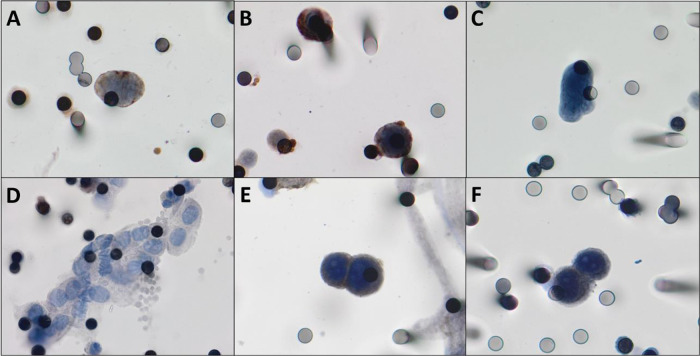
**A,B)** Photomicrographs of CTCs isolated from breast cancer patients immunostained for estrogen receptor and progesterone receptor, respectively (counterstaining with DAB). **C)** CTCs visualized by hematoxylin, without any antibody staining. **D)** One CTM was observed in the filtered blood from a patient with breast cancer. **E,F)** Positive controls, MCF-7 cells “spiked” in healthy blood and stained for estrogen receptor and progesterone receptor respectively. Both images were taken at 400× magnification using a light microscope (Research System Microscope BX61; Olympus, Tokyo, Japan) coupled to a digital camera (SC100; Olympus). **Abbreviations:** CTCs, circulating tumor cells; DAB, 3,3′-daminobenzidine; CTM, circulating tumor microemboli.

CTCs were detected in 22/27 patients (81.5%). The median number of the isolated CTCs was 3.25/mL blood (range, 0-18.6 /mL blood). Figure 2 shows the CTCs and CTMs identified in the study. CTCs were not identified in 5 (18.5%) patients, among whom three had triple-negative breast cancer and two had luminal tumors.

### Protein expression in primary tumors and metastases

The concordance rates for ER, PR, and HER2 expression between primary tumors and metastases were high (Table 2). With respect to changes in the intrinsic subtype that could lead to changes in therapy, we observed no loss of HER2 expression at any metastatic site and a retention of the same pattern in all triple-negative tumors. One patient with a luminal tumor showed an increase in HER2 expression, and one patient with a luminal HER2-positive tumor showed a loss of ER and PR expression (*κ*=0.938, *p*<0.0001; Table 3). Thus, new biopsy findings would have altered the therapeutic approach in only two patients (7.4%), determining the addition of HER2 blockage in one patient and preventing the use of hormone therapy in another patient. In other words, 13.5 biopsies were required to determine one treatment modification.

### Protein expression in CTCs, primary tumors, and metastases

Correlations of protein expression in CTCs with those in primary breast tumors and metastases are shown in Table 4. The number of comparable patients varied depending on the presence of CTCs in immunocytochemical analyses, as previously stated.

The patterns of ER, PR, and HER2 expression in CTCs did not correlate consistently between primary tumors and metastases; wide variations in sensitivity (0%-67% and 0-73%), specificity (67%-94% and 62-95%), and accuracy (PR, 62.5 and 64.7%; ER, 66.7 and 68.4%; HER2, 77.3% and 78.2%) were observed (Table 4). The correlation between CTC and metastasis subtypes was not significant (*κ*=0.156, *p*=0.31; Table S1). The sensitivity, specificity, positive and negative predictive values, and accuracy differed when the metastases and CTCs were classified as triple-negative and non-triple-negative subtypes (Table 5). CTCs determined the metastatic subtype with high specificity (93%), an accuracy of 84.2%, specificity of 50%, a negative predictive value of 88%, and a positive predictive value of 67%.

### Survival

Overall survival was correlated with tumor subtype (*p*=0.008). Patients with primary (ER-positive or -negative) HER2 tumors had a median overall survival duration of 23.7 months; this period was 23.9 months for patients with luminal tumors and 9.5 months for those with triple-negative tumors. The median overall survival durations for metastatic lesion subtypes were 27.5, 23.9, and 9.5 months for HER2, luminal, and triple-negative metastases, respectively (*p*=0.010).

The number of CTCs also correlated with overall survival. Patients without detectable CTC had a median overall survival duration of 9.5 months, whereas this duration was 24.7 months for those with >1 CTC/mL blood (*p*=0.001). Of the seven patients in whom CTCs were not identified, four had luminal and three had triple-negative disease; six died within a median period of 3.3 months (range, 0.8-9.5 months) after blood collection and one patient was lost to follow-up. At the time of blood collection, one of these seven patients had recently been diagnosed with metastasis, three were receiving their first course of metastasis treatment, two were receiving their second course, and one was receiving their fourth course of metastasis treatment.

Of the 16 patients with >1 CTC/mL blood who were not lost to follow-up, 9 died within a median period of 1.1 months (range, 0.3-2.1 months). These patients had a median of 3.5 CTCs/mL blood (range, 1.5-23 CTCs/mL blood); the 7 patients who remained alive had a median of 7.5 CTCs/mL blood (range, 1-23 CTCs/mL blood).

Patients aged ≤34 years had a median overall survival period of 11.8 months, and those aged >35 years had a median overall survival duration of 27.5 months (*p*=0.022).

The median progression-free survival duration correlated with lymph node involvement at the time of diagnosis (N0, 11.5 months; N1, 6.0 months; N2, 5.6 months; *p*=0.036), tumor subtype (triple-negative, 5.8 months; non-triple negative, 10.6 months; *p*=0.020), and metastasis subtype (triple-negative, 5.8 months, non-triple negative, 10.7 months; *p*=0.020). Patients with no detectable CTCs had a median progression-free survival duration of 6.0 months, and those with >1 CTC/mL blood had a median duration of 8.9 months (*p*=0.043). This duration was 6.2 months for patients aged ≤39 years and 10.7 months for those aged >39 years (*p*=0.022).

**Figure 3:**
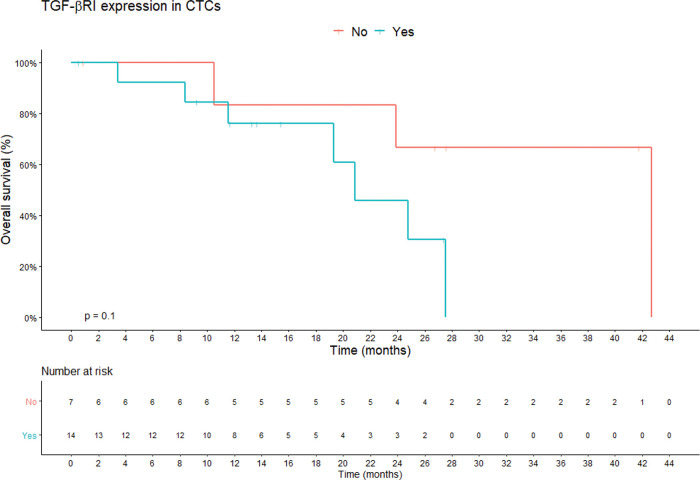
Overall survival according to TGF-β RI expression in CTCs. Shown are the Kaplan-Meier estimates of the overall survival according to TGF-β RI expression in CTCs. According to Kaplan-Meier curve estimates, there is no correlation between TGF-β RI expression in CTCs and overall survival, although the median overall survival of patients without TGF-β RI expression in CTCs was longer than that of patients with TGF-β RI expression (42.6 months *vs.* 20.8 months, respectively; *p*=0.1). *p*-values correspond to the log-rank test used to calculate the difference between survival times in the two patient groups. **Abbreviations:** CTC, circulating tumor cells; TGF-β RI, transforming growth factor-beta receptor I).

**Figure 4:**
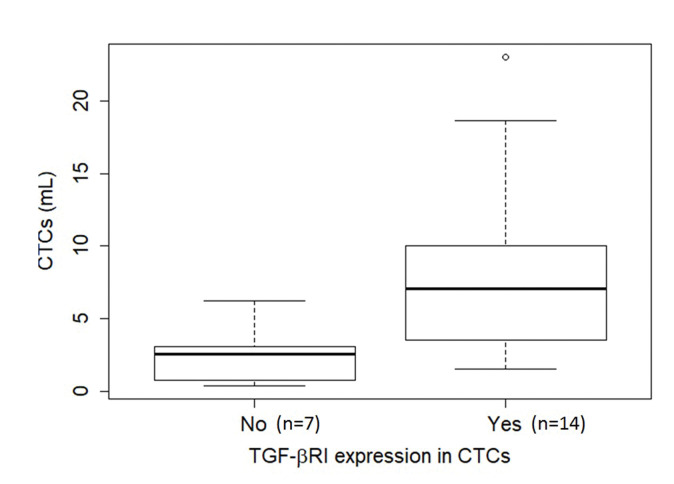
Boxplot of the relationship of CTC abundance (per mL blood) with TGF-β RI expression in CTCs. The median number of CTCs was 7 CTCs/mL blood (range, 1.5-23/mL blood) in the presence of TGF-β RI expression (14 cases) and 2.5 CTCs/mL blood (range, 0.4-6.2/mL blood) in its absence (7 cases), but without statistical significance (*p*=0.09), as determined using the Mann-Whitney U test. Note that in six cases, no CTCs were found in the ISET^®^ spots, hence it was not possible to evaluate TGF-β RI expression. **Abbreviations:** CTCs, circulating tumor cells; TGF-β RI, transforming growth factor-beta receptor I.

TGF-β RI expression in CTCs was not statistically correlated with PFS or overall survival. The median overall survival duration of patients without TGF-β RI expression in CTCs (42.6 months) was much longer than that of patients with TGF-β RI expression in CTCs (20.8 months), but this difference was not significant (Figure 3). TGF-β RI expression seemed to correlate with the median number of CTCs, which was 7 CTCs/mL blood (range, 1.5-23/mL blood) in patients positive for TGF-β RI expression and 2.5 CTCs/mL blood (range, 0.4-6.2/mL blood) in patients that were negative, but without statistical significance (*p*=0.09; Figure 4).

## DISCUSSION

The gradual accumulation of genetic alterations in the primary tumor is thought to alter features that favor metastasis development during cancer evolution (28). Breast cancer metastasis is usually diagnosed based on the clinical symptoms, signs, and radiological findings and is treated according to the characteristics of the primary tumor. Biopsies of suspicious lesions and a confirmation of breast cancer metastasis permit the re-evaluation of the tumor characteristics and HR and HER2 expression patterns. Reported rates between differences in HR and HER2 expression among primary tumors and metastases range from 14.5% to 55.6% for ER and from 0% to 40% for HER2 (29). In a joint analysis of two randomized studies, changes in the ER, PR, and HER2 expression patterns were observed in 12.6%, 31.2%, and 5.5% of metastases, respectively. On average, 7.1 biopsies were required to determine treatment changes because of distinct patterns found in metastases (6)_,_ which was less than what was calculated here in this study.

The metastasis process is complex and involves multiple ordered steps, beginning with the detachment of cells from a primary tumor, followed by the invasion of peritumoral tissues, intravasation in blood or lymph vessels, and leakage through the vascular walls into tissue at a distant site conducive to implantation, and finally new tumor development (30). However, to complete this process, CTCs must evade destruction by the immune system (31). Their circulation in clusters with other tumor cells, platelets, endothelial cells, leukocytes, and/or stromal cells enhances their metastatic capacity relative to isolated cells (32). Several studies have demonstrated the clinical importance of CTCs for predicting metastasis occurrence and tumor evolution, depending on their abundance or their kinetics during treatment (9,10,14,15-17,33). Only a few isolated studies have failed to demonstrate this predictive capacity (34-36).

For some cancer types, such as lung cancer, liquid biopsies are currently used to monitor changes in tumor characteristics during disease evolution. Changes detected at each point of treatment offer valuable information about the response or emergence of new mutations, requiring the need for new tissue biopsies (37). CTCs are detected in approximately 60% of breast cancer cases using the CellSearch^®^ system (38), and CTC data could be used as a surrogate for metastasis biopsy findings if they share the same characteristics. Although there was a higher rate of CTC detection in our sample, the pattern of ER, PR, and HER2 expression on CTCs did not correlate well with those of metastases, being below the desired level to justify the replacement of conventional tissue biopsy by liquid biopsy. It must be considered that the differences in antibodies used may have influenced the results, as the protein expression evaluation in primary tumors and metastases were taken from medical records. However, CTC assessment showed high degrees of specificity and accuracy in metastasis subtype determination between the triple-negative and non-triple-negative tumor groups, despite the small number of cases. From a clinical point of view, treatment is directed to hormone therapy in the presence of HRs; otherwise, chemotherapy is the main treatment of choice (with the use of anti-HER2 agents for tumors overexpressing this protein).

The number of CTCs in primary breast cancer lesions (14-16), after neoadjuvant therapy (39), and in the metastatic setting (13,40) have been shown to correlate with clinical outcomes; however, the presence of CTCs in inflammatory breast cancer specimens after neoadjuvant treatment did not correlate with overall survival (34). Another study revealed no correlation between CTC counts in HER2 tumors treated with targeted therapy and progression free and overall survivals (41), although this finding was not corroborated in other studies in which impacts on both survivals were observed for all breast cancer subtypes (35,42). The kinetics of CTCs during treatment can also be considered predictive of disease progression and death (10). The CellSearch^®^ methodology was used in all of the studies mentioned above. In one study employing the ISET^®^ method, CTCs were identified in 41.2% of initial breast cancer cases (43), but its prognostic evolution was not examined. Studies of CTCs in other tumors, in which the ISET^®^ method has been used, have shown prognostic correlations (44-55). In a retrospective study of 292 patients with metastatic breast cancer, Mego et al. (56) used the Cell Search^®^ method but did not detect CTCs in 35.9% of cases yet observed a positive correlation between this factor and the occurrence of brain metastases and a negative correlation with the occurrence of bone metastases. In such cases, they showed that prognosis was determined by the HR and HER2 status, by the number of metastasis sites, and by the line of treatment, highlighting the heterogeneity of clinical outcomes, although the survival rate was higher for patients with undetectable CTCs than for those with ≥5 CTCs/7.5 mL of blood. Paradoxically, in our study, survival was worse for patients with undetectable CTCs than those positive for CTCs, with no other prognostic factor differentiating these groups. In addition, in patients with at least one CTC detected, those who died had a lower median CTC/mL than those who were alive at the end of the study period. Some of these paradoxical results have been observed by our group for aggressive pancreatic tumors (57) and breast tumors with central nervous system metastases (58), in which patients with CTCs detected via ISET^®^ evolved with a better prognosis. Probably, we can detect any CTC with ISET^®^, including cells that are beyond diverse biological processes, not only active or metastatic cells, but also cells released by primary tumor as garbage or cells that will interact with immune system and come back to the primary tumor, which need more efforts to be better understood. In some situations, such as in the context of established metastasis, CTC counts do not correlate with tumor aggressiveness; other factors (DNA methylation or protein expression of epithelial mesenchymal transition or cytokine secretion) that stimulate tumor progression could be more important. Differential gene expression in tumors can also affect prognosis, altering the expected survival curve through the number of CTCs. For example, one study demonstrated that the survival of patients with breast cancer presenting CTCs with mesenchymal characteristics and no ADAM23 hypermethylation was similar to that of patients with no detectable CTCs and ADAM23 hypermethylation (59).

It is important to consider that most studies regarding CTCs used the EpCAM-based CellSearch^®^ technology instead of the size-based ISET^®^ system. The CellSearch^®^ technique is limited by the lack of CTC detection during EMT, but this factor does not seem to constitute a limitation for tumors in which the presence of EpCAM is strongly associated with tumor progression, such as breast cancer. In these tumors, the loss of EpCAM is gradual and partial, resulting in phenotypic plasticity, enabling the identification of cells with greater metastatic potential (*i.e.*, those with mesenchymal characteristics) despite the maintenance of EpCAM expression (60). In addition, the reduction of EpCAM expression can reduce the size and mass of CTCs, impacting detection by the ISET^®^ method (61). In a direct comparison, more CTCs in breast cancer metastases were identified with CellSearch^®^ than with ISET^®^ (21), likely because of the passage of smaller cells with reduced EpCAM expression through the pores of ISET^®^ membranes (61) or the absence of small cells stained in spots for CTC counting, as described in the Methods section. This hypothesis was evaluated by quantifying EpCAMs in cells in the ISET^®^ filtration eluate, dispensed because of consistent results obtained using ISET^®^ in other tumors (45-49,51,53-55).

Finally, the tumor microenvironment can be considered a system involving multiple cells (e.g., leukocytes, fibroblasts, myoepithelial cells, adipocytes, and endothelial cells), extracellular matrix, soluble factors (e.g., cytokines, growth factors, enzymes, and hormones), and physical factors (e.g., blood pressure, oxygen, and pH) (62). The characteristics of the tumor microenvironment, referred to as “stromal signatures,” can modify the aggressive potential of a tumor and may be determinants of clinical evolution, regardless of the characteristics of the complex interactions between tumors and their microenvironment (63,64).

TGF-β R1-induced EMT enhances cellular migration, and elevated levels of serum TGF-β have been correlated with greater numbers of CTCs in patients with pancreatic and breast tumors (65,66). Similarly, we observed in this study that the expression of TGF-β RI correlated with a greater number of CTCs in patients with breast cancer. We also observed a trend of worse overall survival in cases with TGF-β RI expression in CTCs. This finding was corroborated by the identification of TGF-β RI as an independent prognostic factor in a study involving the ISET^®^ detection of CTCs in patients with head and neck cancer (67). Divella et al. (66) found that the number of CTCs was associated with TGF-β expression and showed that high TGF-β levels were associated with worse prognoses in breast cancer, supporting the finding that TGF-β promotes metastatic tumor growth and invasiveness (23). TGF-β inhibitors may play an important role in chemotherapy or androgen receptor blockade in triple-negative breast cancer (68,69). Galunisertib (LY2157299 monohydrate) is an investigational oral small-molecule inhibitor of the TGF-β receptor I kinase studied either as monotherapy or in combination with other drugs in patients with metastatic cancer (breast, lung, colon, pancreas, hepatocellular cancers and glioblastoma) (70). In a phase 2 study, the combination of galunosertib with gemcitabine in unresectable pancreatic cancer showed increased overall survival compared to treatment with gemcitabine alone (71). In triple-negative breast cancer, there is an ongoing phase 1 clinical trial involving galunisertinib and paclitaxel that will end in 2022 (NCT 02672475) (72).

The limitations of our study must be considered, such as the small sample size, the miscellaneous inclusion of patients, the heterogeneous behavior of the various subtypes of breast cancer, and particularities of the methodology employed (as described above). ISET^®^ may not be the most suitable method for counting CTCs from patients with breast cancer without brain metastases because of its potential failure to identify some cells during EMT and its limited comparability with most other studies (in which EpCAM-based methods have been used). However, it may be taken into consideration when the objective is to evaluate protein expression or gene expression in CTCs, because of the need for manipulating the membranes and storing materials. Future projects employing the ISET^®^ methodology for patients with breast cancer without brain metastases should include research on other factors associated with initial metastasis, such as EpCAM and TGF-β R1, simultaneous analysis of gene expression related to tumor progression, and aspects of the tumor microenvironment and the immune system. Such approaches would provide a more comprehensive scenario, thereby increasing our understanding of the role of CTCs in the progression of various diseases.

## AUTHOR CONTRIBUTIONS

Sanches SM designed the study, performed data analysis and interpretation, and wrote the manuscript. Braun AC performed data analysis and interpretation and the collection and/or assembly of data. Calsavara VF performed data analysis and interpretation. Barbosa PNVP executed the biopsies. Chinen LTD conceived/designed the study, performed data analysis and interpretation, wrote the manuscript, and approved the final version of the manuscript.

## Figures and Tables

**Table 1:** Clinical and pathological characteristics of the patients.

Characteristics	n	%
Number of patients	27	100
Age (years)		
Median	39.0	
Range	26-78	
Tumor subtype		
Luminal HER2-negative	15	55.5
Luminal HER 2-positive	2	7.4
Triple-negative	8	29.6
HER2	2	7.4
Clinical Stage		
I	3	11.1
II	6	22.2
III	12	44.4
IV	6	22.2
Line of treatment		
Treatment-naïve	3	11.1
Adjuvant	18	66.6
Metastatic 1st line	4	14.8
Metastatic 2nd line	1	3.7
Metastatic 3rd line	1	3.7
Site of biopsy		
Lung	11	40.7
Liver	14	51.8
Lymph node	2	7.4

**Table 2:** Estrogen receptor (ER), progesterone receptor (PR), and HER2 receptor expression patterns in primary tumors and metastases.

	ER		PR		HER2	
	metastasis		metastasis		Metastasis	
	+	-	+	-	+	-
**Primary tumor**						
*+*	15	2	10	5	4	0
*-*	0	10	0	12	1	22
concordance	92.5%		81.5%		96.2%	

**Abbreviations:** ER, estrogen receptor; PR progesterone receptor.

**Table 3:** Concordance between primary tumors and metastasis subtypes.*

		Metastasis			Concordance
		Luminal	HER2 or luminal HER2	Triple-negative	
**Primary tumor**	Luminal	14	1	0	93.3%
	HER 2 or luminal HER2	0	4	0	100%
	Triple negative	0	0	8	100%

* *κ*=0.938, *p*<0.0001.

**Table 4:** Comparison of protein expression between CTCs, primary tumors, and metastases.

		Primary tumor						Metastasis					
		ER (n=18)*		PR (n=16)*		HER2 (n=22)*		ER (n=19)*		PR (n=17)*		HER2 (n=23)*	
		-	+	-	+	-	+	-	+	-	+	-	+
**CTC protein expression**	-	4	4	5	4	17	4	5	3	7	3	18	4
	+	2	8	2	5	1	0	3	8	3	4	1	0
Sensitivity		66.7%		55.6%		0		72.7%		57.1%		0	
Specificity		66.7%		71.4%		94.4%		62.5%		70.0%		94.7%	
Positive predictive value		80.0%		71.4%		0		72.7%		57.1%		0	
Negative predictive value		50.0%		55.6%		80.9%		62.5%		70.0%		81.8%	
Accuracy		66.7%		62.5%		77.3%		68.4%		64.7%		78.2%	

**Abbreviations:** CTC, circulating tumor cells; ER, estrogen receptor; PR, progesterone receptor. *Differences in the number of patients are because of the fact that although CTCs were detected in the blood, they were absent in some ISET^®^ spots, as explained in the section of Immunocytochemistry in Materials and Methods.

**Table 5:** Correlation between CTC and metastasis subtypes classified as TN and non-TN.

		Metastasis subtype		
		Triple-negative	Non-triple negative	concordance
**CTC Subtype**	Triple-negative	2	1	66%
	Non-triple negative	2	14	87.5%
Sensitivity		50.0%		
Specificity		93.0%		
Positive predictive value		67.0%		
Negative predictive value		88.0%		
Accuracy		84.2%		

**Abbreviations:** CTC, circulating tumor cells; TN, triple-negative. The difference in the number of patients analyzed (19/27) is because of the fact that although CTCs were detected in the blood, CTCs were absent in some ISET^®^ spots, as explained in the section of Immunocytochemistry in Materials and Methods.

## APPENDIX

**Table S1:** Concordance between CTC and metastasis subtypes.*

		Metastasis subtype		
		Luminal	HER2 Luminal HER2 or	Triple-negative
CTC subtype	Luminal	9	4	2
	HER 2 or luminal Her2	1	0	0
	Triple negative	1	0	2

* *κ*=0.156, *p*=0.313.

**Abbreviations:** CTC, circulating tumor cells.
